# Obeticholic acid ameliorates dyslipidemia but not glucose tolerance in mouse model of gestational diabetes

**DOI:** 10.1152/ajpendo.00407.2018

**Published:** 2019-06-25

**Authors:** Saraid McIlvride, Vanya Nikolova, Hei Man Fan, Julie A. K. McDonald, Annika Wahlström, Elena Bellafante, Eugene Jansen, Luciano Adorini, David Shapiro, Peter Jones, Julian R. Marchesi, Hanns-Ulrich Marschall, Catherine Williamson

**Affiliations:** ^1^School of Life Course Sciences, King’s College London, London, United Kingdom; ^2^Department of Surgery and Cancer, Imperial College London, London, United Kingdom; ^3^Department of Molecular and Clinical Medicine, University of Gothenburg, Gothenburg, Sweden; ^4^National Institute for Public Health and the Environment (RIVM), Bilthoven, The Netherlands; ^5^Intercept Pharmaceuticals, San Diego, California; ^6^School of Biosciences, Cardiff University, Cardiff, United Kingdom

**Keywords:** bile acids, FXR, high-fat diet, insulin resistance

## Abstract

Metabolism alters markedly with advancing gestation, characterized by progressive insulin resistance, dyslipidemia, and raised serum bile acids. The nuclear receptor farnesoid X receptor (FXR) has an integral role in bile acid homeostasis and modulates glucose and lipid metabolism. FXR is known to be functionally suppressed in pregnancy. The FXR agonist, obeticholic acid (OCA), improves insulin sensitivity in patients with type 2 diabetes with nonalcoholic fatty liver disease. We therefore hypothesized that OCA treatment during pregnancy could improve disease severity in a mouse model of gestational diabetes mellitus (GDM). C57BL/6J mice were fed a high-fat diet (HFD; 60% kcal from fat) for 4 wk before and throughout pregnancy to induce GDM. The impact of the diet supplemented with 0.03% OCA throughout pregnancy was studied. Pregnant HFD-fed mice displayed insulin resistance and dyslipidemia. OCA significantly reduced plasma cholesterol concentrations in nonpregnant and pregnant HFD-fed mice (by 22.4%, *P* < 0.05 and 36.4%, *P* < 0.001, respectively) and reduced the impact of pregnancy on insulin resistance but did not change glucose tolerance. In nonpregnant HFD-fed mice, OCA ameliorated weight gain, reduced mRNA expression of inflammatory markers in white adipose tissue, and reduced plasma glucagon-like peptide 1 concentrations (by 62.7%, *P* < 0.01). However, these effects were not evident in pregnant mice. OCA administration can normalize plasma cholesterol levels in a mouse model of GDM. However, the absence of several of the effects of OCA in pregnant mice indicates that the agonistic action of OCA is not sufficient to overcome many metabolic consequences of the pregnancy-associated reduction in FXR activity.

## INTRODUCTION

Gestational diabetes mellitus (GDM) is increasingly prevalent worldwide in association with the rising incidence of obesity in women of reproductive age (13, 17). Affected women have increased risks of hypertensive disorders of pregnancy, including preeclampsia (52), and they have associated dyslipidemia (48) in addition to hyperglycemia (48, 60). There are also risks for the baby, including increased fetal growth that can cause large-for-gestational-age infants and associated shoulder dystocia, birth injury, and neonatal hypoglycemia (3, 7, 23, 45). Furthermore, GDM has long-term consequences for both the mother and offspring, with increased risk of diabetes and other morbidities associated with metabolic syndrome later in life (6, 20).

The nuclear receptor farnesoid X receptor (FXR) not only regulates bile acid homeostasis but also influences lipid and glucose metabolism (27). FXR expression is reduced in rodent models of both type 1 and type 2 diabetes mellitus (16), and *Fxr^−/−^* mice have elevated serum glucose concentrations and insulin resistance (9, 32, 64), as well as hypercholanemia (37, 50). A recent study found that elevated total serum bile acids in the first trimester of pregnancy was associated with increased risk of developing GDM (25). Individual bile acid species also differ between women with GDM and normal pregnancy (19). Another study reported a strong inverse correlation between taurine-conjugated bile acids and glycemic index that enabled discrimination between GDM and uncomplicated pregnancy (15). These changes in bile acid metabolism suggest that FXR activity is altered in GDM. Indeed, one study has reported reduced plasma levels of fibroblast growth factor (FGF) 19 in GDM, which could be indicative of diminished intestinal FXR activation (58). Furthermore, FXR activity is reduced in pregnancy (36), and women with gestational cholestasis have increased rates of GDM (34, 61). We therefore hypothesized that pharmacological activation of FXR could ameliorate the insulin resistance and dyslipidemia that occurs in GDM.

Obeticholic acid (OCA) is a bile acid analog with markedly higher affinity for FXR than the natural ligand, chenodeoxycholic acid (47). OCA is approved for the treatment of primary biliary cholangitis and is in phase 3 studies for the treatment of nonalcoholic steatohepatitis, and clinical studies have shown that OCA administration affects the lipid profile leading to reduced plasma triglycerides (42, 57) and improves insulin sensitivity (41). We hypothesized that OCA treatment could ameliorate impaired glucose tolerance, insulin resistance, and dyslipidemia in a high-fat diet (HFD) mouse model of GDM.

## MATERIALS AND METHODS

### 

#### Animal studies.

Six- to seven-week-old C57BL/6J mice were purchased from Envigo (United Kingdom) and acclimatized to the animal facility for 1 wk. Mice were maintained on a 12-h light-dark cycle with free access to food and water. All experiments were performed in accordance with the Animals (Scientific Procedures) Act 1986 and approved by King’s College London’s Animal Welfare and Ethical Review Body and the Home Office.

Female mice were fed standard CRM diet [normal chow (NC)] or a high-fat diet (HFD; RM AFE 60% fat, cat. no. 824054, Special Diets Services, UK), for 4 wk before being mated with age-matched male mice (see Table 1 for diet composition). Upon identification of a copulatory plug, considered to be *day 1* of pregnancy (GD1), the females either continued the same diet or received a diet supplemented with 0.03% OCA (~50 mg/kg per day). This dose was based on a previous study of OCA administration in mice (4). A high dose was chosen due to the short window of treatment allowed by pregnancy. Diet-matched virgin female mice (D0) were used as nonpregnant controls and received OCA-supplemented diets for the same period of time as pregnant mice. Mice were euthanized by CO_2_ inhalation on GD18 (or equivalent) after fasting for 4 h from 8 AM and blood and tissues were collected.

**Table 1.** Diet composition

Composition [% (wt/wt)]	CRM (Normal Chow)	High-Fat Diet
Water	10.0	4.09
Crude protein	18.35	25.89
Vegetable-derived fat	3.36	4.87
Animal-derived fat	0.0	29.25
Crude fiber	4.23	4.98
Ash	6.27	4.8
Nitrogen-free extract	57.39	25.03
Energy		
AFE (kcal AFE/g)	3.33	5.13
AFE from fat (% kcal)	9	60
AFE from protein (% kcal)	22	20
AFE from carbohydrate (% kcal)	69	20

AFE, Atwater fuel energy; CRM, standard diet.

#### Glucose and insulin tolerance tests.

Glucose and insulin tolerance tests were performed on GD16 or GD17, respectively. Mice were fasted for 6 h from 8 AM and administered either 2 g/kg body weight glucose or 0.75 IU/kg body weight insulin by intraperitoneal injection. Blood glucose concentrations were measured using a FreeStyle Lite glucometer (Abbott Healthcare, UK).

#### mRNA expression analysis.

Total RNA was isolated from frozen tissue samples using the RNeasy Mini Kit (Qiagen, UK) and reverse transcribed using Superscript II Reverse Transcriptase (Thermo Fisher Scientific, UK) according to the manufacturer’s instructions. Gene expression was quantified by real-time PCR using SYBR Green Mastermix (Sigma-Aldrich, UK) and a Viia7 system (Life Technologies, UK). Cyclophilin b was used as a housekeeping gene, and relative expression of target genes was calculated by the ∆∆Ct method. The relative change in expression is given as 2^-∆∆Ct^. Primer sequences are provided in Table 2.

**Table 2.** Primer sequences

Gene	Forward Primer (5′-3′)	Reverse Primer (3′-5′)
*Abcg5*	TCAATGAGTTTTACGGCCTGAA	GCACATCGGGTGATTTAGCA
*Abcg8*	TGCCCACCTTCCACATGTC	ATGAAGCCGGCAGTAAGGTAG
*Asbt*	TCCTGGCTAGACTAGCTGGTC	CTGAGTGTTCTGCATTCCAGTT
*Bsep*	AAGCTACATCTGCCTTAGACAC	CAATACAGGTCCGACCCTCTCT
*Cd68*	CCACAGGCAGCACAGTGGACA	GCAGAAGCTTTGGCCCAAGGG
*Cyp7a1*	AGCAACTAAACAACCTGCCAGTACTA	GTCCGGATATTCAAGGATGCA
Cyclophilin B	TGGAGAGCACCAAGACAGACA	TGCCGGAGTCGACAATGAT
*Cyp8b1*	TAGCCCTCTTTCCTCCACTCAT	GAACCGATCGAACCTAAATTC
*Fgf15*	GAGGACCAAAACGAACGAAAT	ACGTCCTTGATGGCAATCG
*Fgfr4*	CGCCAGCCTGCTACTATACAAA	CCAGAGGACCTCGAGTCCAA
*Hmgcr*	TTGGCACCATGTCAGGCGTCC	AGCGACACACAGGCCGGGAA
*Ibabp*	TGAGAGTGAGAAGAATTACGA	TTACGTCCCCTTTCAATCACG
*Ldlr*	CTGGTGACCGAAAACATCCAGT	AATCAACCCAATAGAGACGGCC
*Ntcp*	GAAGTCCAAAAGGCCACACTATTGT	ACAGCCACAGAGAGGGAGAAAAG
*Ostα*	CTGAGCATAGTGGGCCCTGTTC	AGCCTGGCGCTCTTCCTCAGAAATT
*Ostβ*	TGACAAGCATGTTCCTCCTCAG	TTCTTTGTCTTGTGCCTGCTTC
*Shp*	CGATCCTCTTCAACCCAGATG	AGGGCTCCAAGACTTCACACA
*Srb1*	GGGAGCGTGGACCCTATGT	ACACGGTGTCGTTGTCATTGA
*Srebp2*	CCTAGACCTCGCCAAAGGTG	AGGCTGTAGCGGATCACAT
*Tnfα*	AGCCCACGTCGTAGCAAACCA	CCGTTGGCCAGGAGGGCGTT
β-klotho	GATGAAGAATTTCCTAAACCAGGTT	AGCCTGGCGCTCTTCCTCAGAAATT

#### Plasma glucagon-like peptide-1 measurement.

Glucagon-like peptide (GLP)-1 was measured in plasma samples taken from the portal vein at euthanization on GD18 or equivalent by GLP-1 (Active) ELISA (Millipore, UK) according to the manufacturer’s protocol.

#### Lipid biochemistry.

Lipids were extracted from tissues in a 0.125 M potassium phosphate buffer and normalized to total protein content. Cholesterol, LDL cholesterol, HDL cholesterol, triglycerides, free fatty acids, and total protein were measured in plasma and tissue extracts using a Unicel DxC 800 autoanalyzer (Beckman-Coulter, the Netherlands) and dedicated kits, with the exception of free fatty acids, which were measured using a kit from Wako Diagnostics (Germany).

#### Bile acid quantification.

Bile acids were measured in plasma and cecal samples collected at euthanization on GD18 or equivalent using a high-performance liquid chromatography Alliance 2695 system coupled to a Xevo TQ mass spectrometer using a SunFire C18 column (4.6 × 100 mm, 3.5 μm; Waters, UK), as previously described (49).

#### 16S rRNA gene sequencing.

DNA was extracted from cecal samples using the QIAamp Fast DNA Stool Mini Kit (QIAGEN, UK) according to the manufacturers’ instructions. Sample libraries were prepared as previously described (35). Sequencing was performed using the MiSeq Reagent Kit v3 and paired-end 300 bp chemistry on an Illumina Miseq platform (Illumina). Data analysis was performed using Mothur software (v1.35.1; https://www.mothur.org/) following the MiSeq SOP Pipeline (54). Sequence alignments were obtained from the Silva bacterial database (https://www.arb-silva.de/), and sequences were classified according to the Ribosomal Database Project reference sequence files using the Wang method (18). Nonmetric multidimensional scaling plots and permutational multivariate analysis of variance *P* values were produced using the UniFrac weighted distance matrix created by Mothur, and analysis was carried out using the Vegan library (5) within the statistical software, R (www.r-project.org). Bacterial relative abundance was expressed as extended error bar plots using the Statistical Analysis of Metagenomic Profiles software package using White’s nonparametric *t*-test with Benjamini-Hochberg false discovery rate. *P* and q values < 0.05 were considered significant.

#### Statistical analysis.

Data are expressed as means ± SE. Statistical analysis was performed using GraphPad Prism software (version 7; GraphPad Software), unless otherwise specified. Data were assessed for normality using the Shapiro-Wilk normality test. Where two groups were being compared, Student’s *t*-test was used. For comparison of three or more groups, ANOVA was used, followed by Tukey’s post hoc analysis, unless otherwise specified. A *P* value < 0.05 was considered significant.

## RESULTS

### 

#### OCA ameliorates weight gain and white adipose tissue inflammation in nonpregnant HFD-fed mice.

**Fig. 1.**
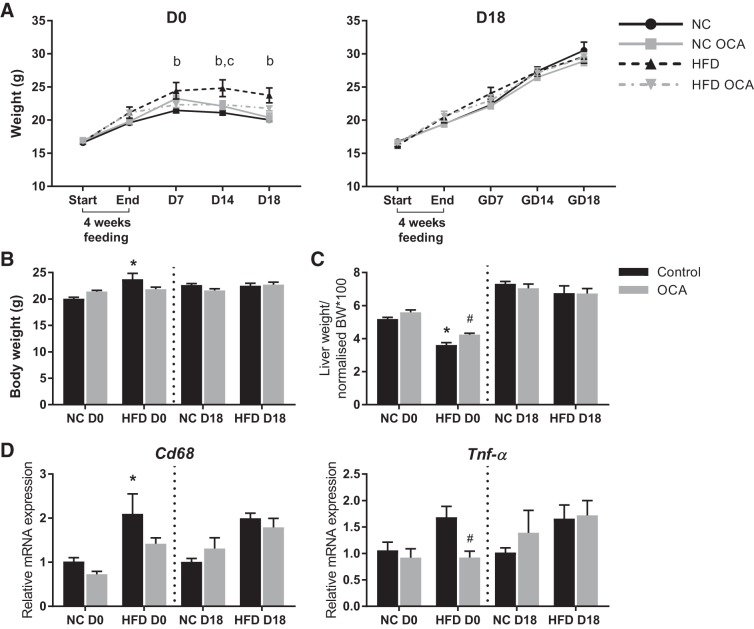
Obeticholic acid (OCA) ameliorates weight gain and white adipose tissue inflammation in nonpregnant high-fat diet (HFD)-fed mice. *A*: weight gain in nonpregnant (D0) and pregnant (D18) mice before and after HFD prefeeding and at *gestational days* (GD) *7*, *14*, and *18*. *P* < 0.05 as determined by two-way ANOVA followed by Tukey’s multiple comparisons test for the following comparisons: b, normal chow (NC) vs. HFD; c, HFD vs. HFD OCA. *B*: body weight (BW) at GD18, without uterine horn. *C*: liver weight at GD18 normalized to BW without uterine horn. *D*: relative mRNA expression of inflammatory markers in gonadal white adipose tissue at GD18. *P < 0.05 vs. NC control, #*P* < 0.05 vs. HFD control as determined by one-way ANOVA followed by Tukey’s multiple comparisons test. Data are expressed as means ± SE; *n* = 5–11 mice per group.

Nonpregnant mice that were treated with OCA gained significantly less weight than mice fed HFD alone; however, no difference was observed in pregnant mice (Fig. 1*A*). This difference was also evident when maternal weight was normalized by subtracting the weight of the uterine horn and contents at GD18 (Fig. 1*B*). The liver-to-body weight ratio was significantly reduced by HFD feeding in nonpregnant mice only, which was also lessened by OCA treatment (Fig. 1*C*). OCA also significantly reduced the expression of inflammatory markers *Cd68* and *Tnf-α* in white adipose tissue of nonpregnant HFD-fed mice (Fig. 1*D*).

#### OCA does not improve glucose tolerance in HFD-fed mice.

**Fig. 2.**
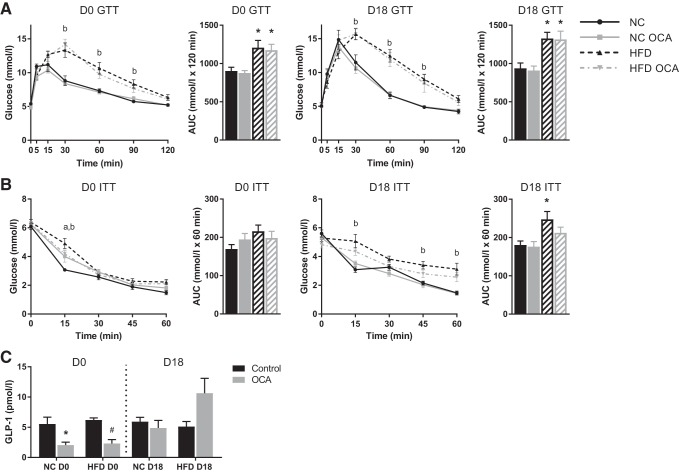
Obeticholic acid (OCA) does not improve glucose tolerance in high-fat diet (HFD)-fed mice. *A*: glucose tolerance tests (GTT) were performed on *day 16* of pregnancy [or equivalent for nonpregnant (D0) controls]; *n* = 7–10 mice per group. *B*: insulin tolerance tests (ITT) were performed on *day 17* of pregnancy (or equivalent for D0 controls); *n* = 7–11 mice per group. *P* < 0.05 as determined by two-way repeated measures ANOVA followed by Tukey’s multiple comparisons test; a, normal chow (NC) vs. NC OCA; b, NC vs. HFD. For area under the curve (AUC), *P < 0.05 vs. NC, as determined by one-way ANOVA followed by Tukey’s multiple comparisons test. *C*: glucagon-like peptide 1 (GLP-1) concentrations were measured in plasma collected from the portal vein upon euthanization at *gestational day* *18* (D18) (or equivalent for D0 controls). *P < 0.05 vs. NC, #*P* < 0.05 vs. HFD, as determined by one-way ANOVA followed by Tukey’s multiple comparisons test; *n* = 6–8 mice per group. Data are expressed as means ± SE.

Glucose and insulin tolerance tests were performed on GD16 or GD17, respectively, or equivalent for nonpregnant controls. Both nonpregnant and pregnant mice displayed significantly impaired glucose tolerance upon HFD feeding (Fig. 2*A*), but only pregnant HFD-fed mice had significantly reduced insulin tolerance (Fig. 2*B*). There was no effect of OCA treatment on glucose tolerance. Insulin tolerance was improved by OCA in pregnant HFD-fed mice, but it did not return to baseline levels. OCA significantly reduced fasted plasma GLP-1 levels in NC- and HFD-fed nonpregnant mice, but not in pregnant mice (Fig. 2*C*).

#### OCA reduces plasma cholesterol in nonpregnant and pregnant mice.

**Fig. 3.**
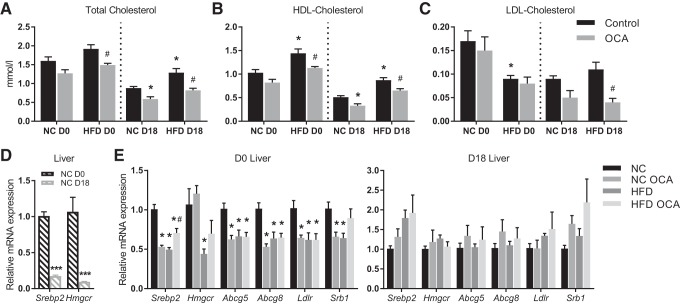
Obeticholic acid (OCA) reduces plasma cholesterol in nonpregnant and pregnant mice. *A*–*C*: maternal plasma concentrations of total cholesterol, HDL cholesterol, and LDL cholesterol on *day 18* of pregnancy (D18) [or equivalent for nonpregnant (D0) controls]; *n* = 5–7 mice per group. *P < 0.05 vs. normal chow (NC), #*P* < 0.05 vs. high-fat diet (HFD), within D0 or D18 groups, as determined by one-way ANOVA followed by Tukey’s multiple comparisons test. *D*: mRNA expression of lipid homeostasis genes in maternal liver at D18 relative to D0; *n* = 4–6 mice per group. ***P < 0.001 as determined by Student’s *t*-test. *E*: mRNA expression of lipid homeostasis genes in maternal liver at D18 (or equivalent); *n* = 4–6 mice per group. *P < 0.05 vs. NC, #*P* < 0.05 vs. HFD, within D0 or D18 groups, as determined by one-way ANOVA followed by Tukey’s multiple comparisons test.

HFD feeding caused a significant increase in total plasma cholesterol in pregnant mice only. However, OCA administration significantly reduced the total cholesterol in HFD-fed nonpregnant mice (Fig. 3*A*). In pregnant mice, OCA treatment significantly reduced total cholesterol in both NC- and HFD-fed mice (Fig. 3*A*). This effect was largely due to a reduction in HDL cholesterol, although OCA also significantly reduced LDL cholesterol in HFD-fed pregnant mice (Fig. 3, *B* and *C*). There was no effect of OCA on plasma triglyceride or free fatty acid levels (data not shown), and hepatic lipid levels were also unaffected by OCA treatment (data not shown). When expression of lipid homeostasis targets was assayed in maternal liver, the analysis showed that in NC-fed mice OCA significantly reduced the expression of targets, including *Srebp2*, a transcription factor regulating cholesterol synthesis; *Abcg5*/*8*, which effluxes sterols (including cholesterol) into bile; and the LDL and HDL receptors, *Ldlr* and *Srb1*. Expression of *Hmgcr*, the rate-limiting enzyme in cholesterol synthesis, was unchanged by OCA treatment. However, these effects were not apparent in pregnant mice (Fig. 3*E*), despite them also having reduced plasma cholesterol. This observation led us to conclude that either the reduction in cholesterol is not due to any transcriptional changes in hepatic lipid homeostasis pathways or the mechanisms are distinct in nonpregnant and pregnant mice. Consistent with previous studies (43), mRNA expression of cholesterol homeostasis targets is reduced in late pregnancy in mice (Fig. 3*D*). Furthermore, HFD feeding also reduced expression of these targets, with no further effect of OCA with the exception of increased *Srebp2* expression in HFD-fed nonpregnant mice. This suggests that the repression of these targets by pregnancy and HFD has abrogated any impact of OCA. There was no effect of OCA on lipid biochemistry in the placenta or fetal liver (data not shown).

#### OCA modulates plasma bile acids in NC- but not HFD-fed mice.

**Fig. 4.**
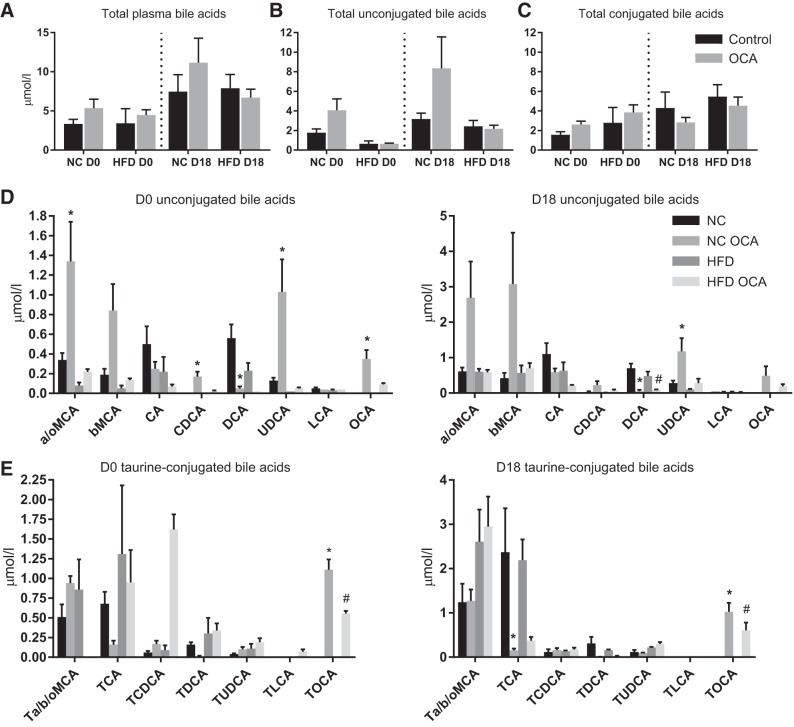
Obeticholic acid (OCA) alters serum bile acids in normal chow (NC) but not high-fat diet (HFD)-fed mice. Maternal plasma concentrations of total bile acids (*A*), total unconjugated bile acids (*B*), total conjugated bile acids (*C*), and individual bile acid species (*D*–*E*) are shown; *n* = 3–6 mice per group. *P < 0.05 vs. NC, #*P* < 0.05 vs. HFD, as determined by one-way ANOVA followed by Tukey’s multiple comparisons test. a/b/oMCA, α/β/ω-muricholic acid; CA, cholic acid; CDCA, chenodeoxycholic acid; D0, nonpregnant mice; D18, pregnant mice; DCA, deoxycholic acid; UDCA, ursodeoxycholic acid; LCA, lithocholic acid; OCA, obeticholic acid; T, taurine; TCA, taurocholic acid.

Because of the key role of FXR in modulating bile acid homeostasis, bile acid concentrations were measured in maternal plasma. There was no significant effect of HFD or OCA on either total plasma bile acids or total unconjugated and conjugated bile acids (Fig. 4, *A*–*C*). HFD did not significantly impact individual bile acid species concentrations (Fig. 4, *D* and *E*). In nonpregnant NC-fed mice, OCA caused an increase in α- and ω-muricholic acid (MCA), chenodeoxycholic acid (CDCA), and ursodeoxycholic acid (UDCA) and a decrease in deoxycholic acid (DCA). OCA did not alter any bile acid species in HFD-fed nonpregnant mice. In pregnancy, OCA treatment similarly caused a significant increase in UDCA and decrease in DCA and also caused a decrease in taurocholic acid (TCA) in NC-fed mice. However, in HFD-fed mice, OCA decreased DCA concentrations only. Of note, OCA and tauro-OCA were both present in plasma at similar levels across all groups fed OCA, confirming absorption of OCA from the diet.

#### OCA affects cecal bile acids disparately in pregnant and nonpregnant mice.

**Fig. 5.**
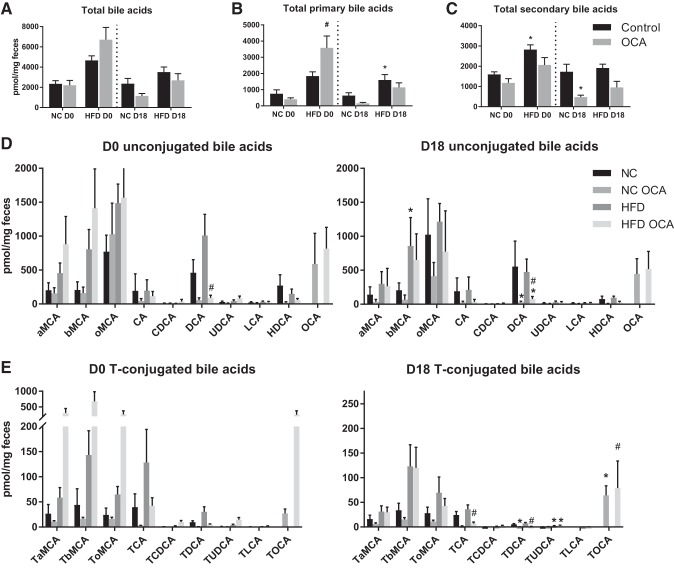
Obeticholic acid (OCA) affects cecal bile acids disparately in pregnant (D18) and nonpregnant (D0) mice. Cecal concentrations of total bile acids (*A*), total primary bile acids (*B*), total secondary bile acids (C), and individual bile acid species (*D*–*E*) in D0 and D18 mice are shown; *n* = 5–6 mice per group. *P < 0.05 vs. normal chow (NC), #*P* < 0.05 vs. high-fat diet (HFD), as determined by one-way ANOVA followed by Tukey’s multiple comparisons test. a/b/oMCA, α/β/ω-muricholic acid; CA, cholic acid; CDCA, chenodeoxycholic acid; DCA, deoxycholic acid; UDCA, ursodeoxycholic acid; LCA, lithocholic acid; HDCA, hyodeoxycholic acid; T, taurine; TCA, taurocholic acid.

Bile acids were also measured in the cecum, which reflects the bile acid composition of the colon and feces (49). Total cecal bile acid concentrations were not different between any of the groups (Fig. 5*A*). However, HFD and OCA affected total primary and secondary bile acids disparately in nonpregnant and pregnant mice (Fig. 5, *B* and *C*). HFD feeding caused an increase in the total concentration of primary bile acids in D18 mice and also elevated total secondary bile acids in D0 mice. OCA caused an increase in total primary bile acids in HFD-fed D0 mice, predominantly due to raised conjugated and unconjugated MCA (Fig. 5, *D* and *E*). However, in pregnant mice, OCA reduced total secondary bile acids in NC-fed D18 mice, largely due to reduced DCA and ωMCA (Fig. 5*D*). As expected, cecal bile acids were predominantly unconjugated (Fig. 5, *D* and *E*).

#### OCA alters cecal microbiome in NC- but not HFD-fed mice.

**Fig. 6.**
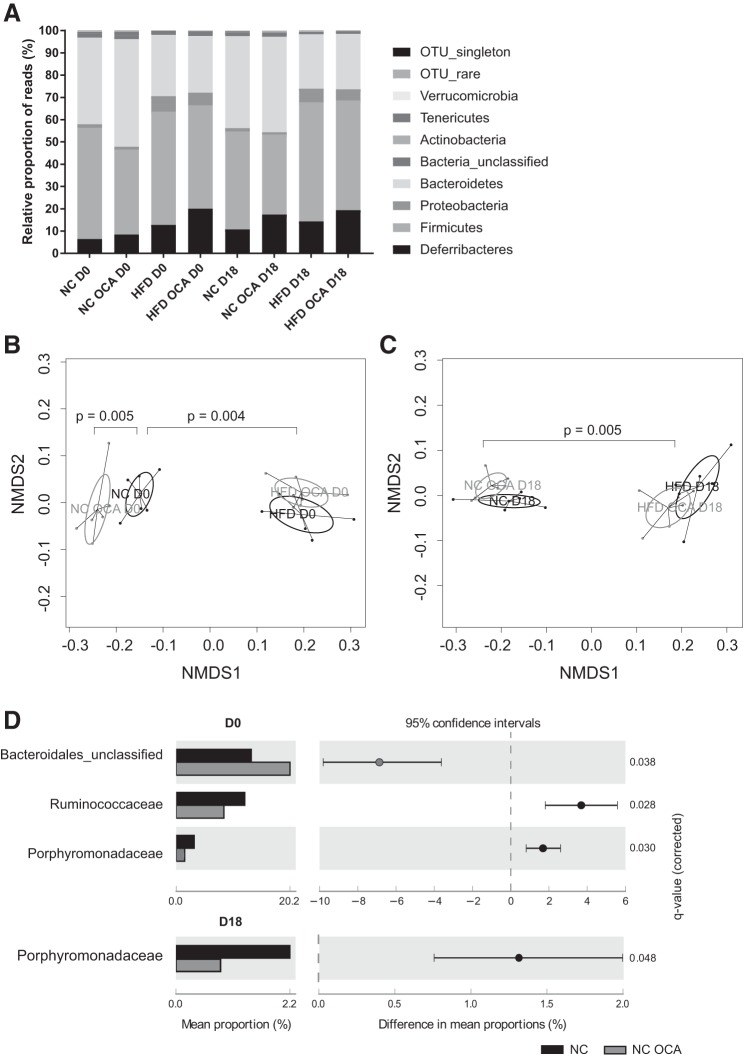
Obeticholic acid (OCA) alters cecal microbiome in normal chow (NC) but not high-fat diet (HFD)-fed mice. Microbiota were measured in cecal samples from nonpregnant (D0) and pregnant (D18) mice using 16S rRNA gene sequencing. *A*: relative proportion of reads according to bacterial phyla. *B*: nonmetric multidimensional scaling (NMDS) plot with permutational multivariate analysis of variance (PERMANOVA) *P* values showing dissimilarities between cecal microbiota of D0 mice. Comparisons shown are NC D0 vs. NC OCA D0 and NC D0 vs. HFD D0. *C*: NMDS plot with PERMANOVA *P* values showing dissimilarities between cecal microbiota of D18 mice. Comparison shown is NC D18 vs. HFD D18. *D*: bacterial families of significantly different relative abundance in D0 (upper panel) and D18 (lower panel) NC and NC OCA mice. Analyzed using White’s nonparametric *t*-test with Benjamini-Hochberg false discovery rate; *n* = 5–6 mice per group.

Because of the relationship between bile acids and the intestinal microbiome (56), 16S rRNA genes were sequenced to characterize the cecal microbiota. Distribution of cecal microbiota was most affected by HFD feeding (Fig. 6*A*), with alterations in *Bacteroidetes*, *Proteobacteria*, and *Deferribacteres* in particular. Similarly, this is reflected in nonmetric multidimensional scaling plots showing that the composition of cecal microbiota is markedly different in HFD-fed mice compared with NC-fed mice (Fig. 6, *B* and *C*). However, no effect of OCA was observed on bacterial taxa in the HFD groups (data not shown). In NC-fed nonpregnant mice, however, OCA treatment led to distinct differences in microbiota (Fig. 6*B*). Furthermore, OCA had a greater impact on relative abundance of bacterial families in nonpregnant mice compared with pregnant mice (Fig. 6*C*). In particular, OCA treatment caused a large increase in an unclassified *Bacteroidales* family and a decrease in the *Firmicutes* family *Ruminococcaceae*.

#### Both pregnancy and HFD alter OCA’s impact on FXR signaling.

**Fig. 7.**
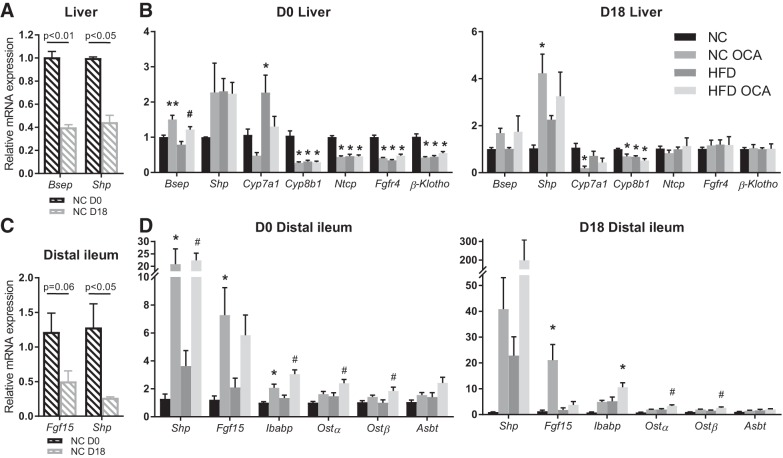
Effect of obeticholic acid (OCA) on expression of bile acid homeostasis genes is altered by pregnancy. Relative mRNA expression of targets in farnesoid X receptor signaling pathways in maternal liver and distal ileum. *A*: hepatic gene expression in pregnant mice (D18) relative to nonpregnant mice (D0). Data were analyzed by unpaired Student’s *t*-test; *n* = 5–6 mice per group. *B*: hepatic gene expression in D0 and D18 mice. *P < 0.05 vs. normal chow (NC), **P < 0.01 vs. NC, #*P* < 0.05 vs. high-fat diet (HFD), as determined by one-way ANOVA followed by Tukey’s multiple comparisons test; *n* = 5–6 mice per group. *C*: distal ileal gene expression in D18 relative to D0 mice. Data were analyzed by unpaired Student’s *t*-test; *n* = 5–6 mice per group. *D*: distal ileal gene expression in D0 and D18 mice. *P < 0.05 vs. NC, **P < 0.01 vs. NC, #*P* < 0.05 vs. HFD, as determined by one-way ANOVA followed by Tukey’s multiple comparisons test; *n* = 5–6 mice per group.

Our data show that several effects of OCA in nonpregnant mice are absent in pregnant mice. Gene expression of FXR targets was therefore examined in the liver and distal ileum. Confirming previous studies from our group (36, 44) and others (40), expression of FXR targets in the liver and distal ileum is reduced in pregnancy (Fig. 7, *A* and *C*). OCA treatment caused induction of *Bsep* and reduction of *Cyp8b1*, *Ntcp*, *Fgfr4*, and β-klotho in the livers of nonpregnant NC-fed mice (Fig. 7*B*). In pregnant mice, there was induction of *Shp* and reduction of *Cyp7a1* and *Cyp8b1*. In the ileum, OCA increased *Shp*, *Fgf15*, and *Ibabp* in nonpregnant NC-fed mice, but only *Fgf15* was significantly altered in pregnant NC-fed mice (Fig. 7*D*). OCA treatment in HFD-fed mice had minimal impact on gene expression in the liver, with only *Bsep* increased in nonpregnant mice (Fig. 7*B*). In the ileum, *Shp*, *Ibabp*, and *Ostα/β* were all induced by OCA in nonpregnant mice, but only *Ostα/β* was increased in pregnant HFD-fed mice (Fig. 7*D*).

## DISCUSSION

Our data show that OCA improves dyslipidemia and insulin resistance but has no impact on impaired glucose tolerance in a mouse model of GDM. Many of the effects of OCA that were observed in nonpregnant mice were not seen in pregnancy, consistent with the known reduction in FXR activity in pregnancy (36); thus, the efficacy of OCA was limited.

To induce features of gestational diabetes, mice were fed a high-fat diet (HFD) for 4 wk before and during pregnancy. This model has previously been used to induce features of GDM in mice, such as insulin resistance and dyslipidemia (24, 26, 31). A period of only 4 wk of HFD exposure before pregnancy is not sufficient to cause a diabetic phenotype; however, continued feeding throughout pregnancy leads to progressive glucose intolerance and insulin resistance, mimicking human disease. In our model, the mice receiving a HFD were significantly glucose intolerant but only demonstrated significant insulin resistance when coupled with pregnancy. This difference could explain why OCA had no effect on insulin sensitivity in nonpregnant animals, in contrast to previous animal and human studies (41, 55). Alternatively, it could be due to the duration of OCA treatment, which is limited in our model by the 19–21 day gestation length in mice. Although insulin resistance was lessened in pregnant HFD-fed OCA mice, there was no effect of OCA on glucose tolerance. Furthermore, OCA treatment reduced fasting plasma GLP-1 in both NC- and HFD-fed nonpregnant mice. The involvement of FXR in GLP-1 secretion is unclear, with reports of FXR activation both increasing and inhibiting GLP-1 secretion (46, 53). Nevertheless, this effect was not apparent in pregnant mice, suggesting that OCA interacts differently with FXR in pregnancy.

OCA did consistently impact plasma cholesterol levels, as total cholesterol was significantly decreased in nonpregnant HFD-fed mice and pregnant NC- and HFD-fed mice treated with OCA. This change was due to decreased HDL and LDL cholesterol in pregnant HFD-fed mice. This finding has previously been observed in nonpregnant animals (14, 62) and has been suggested to be due to increased reverse cholesterol transport. Xu et al. (62) observed increased fecal cholesterol and reduced fecal bile acids, whereas Dong and colleagues (14) observed increased mRNA and protein expression of hepatic scavenger receptor class B type 1 (SR-B1) accompanied by increased fecal cholesterol. Fecal cholesterol concentrations were not measured in this study. However, we did not observe any changes in fecal bile acids with OCA treatment. Interestingly, our data show that hepatic *Srb1* expression was reduced in nonpregnant NC-fed mice treated with OCA, a group with no changes in plasma cholesterol, but it was not altered in pregnant animals and there was no impact of OCA treatment in HFD-fed mice.

OCA significantly reduced weight gain at D14 in nonpregnant HFD-fed mice, as has been observed previously (22). This effect was not apparent in pregnant animals and is likely due to the gestational changes that impact maternal morphometry, such as hyperphagia and promotion of storage of energy in white adipose tissue, which have a greater impact on body weight than HFD. HFD is commonly used to induce maternal obesity in rodents, but the prepregnancy exposure period is typically longer than 4 wk (21, 26, 28, 30). Adipose depot mass was not measured in our study; therefore, it is possible that OCA may have caused differences without affecting total body weight, as has been observed in HFD-fed rabbits treated with OCA (33). As well as reducing weight gain, OCA also reduced mRNA expression of inflammatory markers in white adipose tissue of HFD-fed nonpregnant mice. FXR has been shown to regulate inflammatory responses via nuclear factor κB (59), and OCA has previously been shown to reduce expression of inflammatory markers in adipose tissue (22). Notably, this effect was not observed in pregnant mice. It is known that expression of proinflammatory adipokines is increased in white adipose tissue in rodent pregnancy (8, 10, 63); therefore, it is possible that the gestational signals that mediate this proinflammatory profile are not overcome by FXR activation by OCA.

OCA also impacted cecal bile acids differently in pregnant and nonpregnant mice. In plasma, the effect of OCA on individual bile acids generally followed the same pattern in pregnant and nonpregnant mice, whereas in the cecum OCA caused a significant increase in the total concentration of primary bile acids in nonpregnant HFD-fed OCA mice. In pregnancy, however, OCA caused a significant decrease in the total concentration of secondary bile acids in NC-fed OCA mice. There is a trend for primary bile acids to also be reduced in the cecum from this group, as well as increased plasma unconjugated bile acids, which could suggest increased absorption of bile acids in the ileum.

Because of the alterations in cecal bile acid concentrations, we investigated whether OCA-treated mice had altered microbiota in the cecum. Feeding mice HFD caused the most marked changes in the cecal microbiota, which could potentially mask any effect of OCA treatment in these groups. OCA treatment of NC-fed mice also caused significant differences in microbiota that were not observed in pregnant mice, such as significantly reduced *Ruminococcaceae*, which are a family of *Firmicutes* bacteria, and a significant increase in an unclassified family of the *Bacteroidetes* phylum. This is consistent with a recent study from our group showing that mice fed CA, an FXR ligand albeit significantly less potent than OCA, have an increased ratio of *Bacteroidetes* to *Firmicutes* accompanied by enhanced enterohepatic feedback via *Fgf15* (44). These large changes were absent in pregnant mice, highlighting the different effect of OCA on the microbiome in pregnant and nonpregnant mice. However, it was surprising that there were not more differences between microbiota of nonpregnant and pregnant control mice, which is in contrast to our recent study that found that pregnancy, as well as CA feeding, is associated with an increased ratio of *Bacteroidetes* to *Firmicutes*. We observed a small trend for an increased *Bacteroidetes*-to-*Firmicutes* ratio, but it did not reach significance. This may be explained by the fact that the Ovadia study (44) used whole shotgun genome metagenomic sequencing; additionally, the mice were bred and housed differently and received a different control diet (RM3).

Several of the effects of OCA described herein were not evident in pregnant mice. The similar plasma and cecal levels of OCA and tauro-OCA across all of the groups suggest that this is not due to differences in OCA intake or absorption. A potential explanation for the absence of OCA-related effects in pregnancy could have been higher circulating amounts of FXR antagonistic bile acids, such as taurine-conjugated α/β-MCA (49); however, our serum data do not support this explanation. Hepatic FXR activity is known to be reduced in pregnancy (36) due to the effects of reproductive hormones including progesterone sulphates (1) and 17β-estradiol (51). Ileal expression of FXR targets is also known to be reduced in pregnancy (39, 44). Therefore, it is plausible that activation of FXR by OCA is diminished in pregnancy, and this decreased activation is why effects such as decreased white adipose tissue inflammation and reduced GLP-1 secretion are not seen. It should also be noted that induction of FXR targets by OCA differed between NC- and HFD-fed pregnant mice; in particular, there was no increase in ileal *Fgf15* and corresponding decrease in *Cyp7a1* in the liver of HFD-fed OCA pregnant mice. It has been observed previously in male C57BL/6 mice that *Fgf15* expression was decreased in the distal ileum after 4–8 wk of HFD (12), and decreased circulating FGF19 has been reported in human obese subjects (2). We observed no impact of HFD alone on FXR targets in the ileum; nevertheless, it is possible that HFD has an independent effect on enterohepatic feedback via FGF15 that should be taken into consideration. Despite these differences, the effect of OCA on plasma cholesterol is consistent between nonpregnant and pregnant mice and was also evident in HFD-fed mice. The mechanism for the reduction in plasma cholesterol is unclear, as expression of genes involved in hepatic lipid homeostasis was unchanged by OCA treatment in pregnant mice; therefore, future studies are needed.

A limitation of this study is the relatively mild disease phenotype induced by the HFD. Although pregnant HFD-fed mice were significantly insulin resistant compared with pregnant NC-fed mice, fasting glucose and plasma triglyceride levels were normal. We were unable to monitor food intake due to the consistency of the HFD, and a recent study has shown that consumption of HFD can vary substantially in C57BL/6J mice (11). It would therefore be of interest to also study a genetic model of GDM, such as the heterozygous leptin receptor deficient (Lepr(db/+)) mouse (29). Although we cannot be certain that food intake was similar between groups, it is known that OCA supplementation of HFD did not affect food intake in transgenic mice bred on a C57BL/6J background (38). Another limitation of using HFD is that it is manufactured with purified ingredients, unlike the control NC. Although we are not specifically interested in the effects of OCA on a HFD in pregnancy but rather the disease features it induces, it could be a confounding factor to take into consideration.

In conclusion, OCA does not improve glucose tolerance in a HFD model of GDM. However, OCA treatment significantly improved maternal hypercholesterolemia. It is likely that FXR activation by OCA is blunted in pregnant mice due to the gestational suppression of FXR activity. Therefore, gestational suppression of FXR activity should be taken into account when considering FXR agonists as a treatment for metabolic disorders in pregnancy.

## GRANTS

This work was supported by the Wellcome Trust (Grant no. P30874), Lauren Page Trust, Tommy’s Charity, Guy’s and St. Thomas’ Charity, and the National Institute of Health Research (NIHR) Biomedical Research Centre (BRC) at Guy’s and St. Thomas’ National Health Service (NHS) Foundation Trust. Both J. R. Marchesi and J. A. K. McDonald, at Imperial College London, receive financial support from the NIHR BRC based at Imperial College Healthcare NHS Trust and Imperial College London.

## DISCLAIMERS

The views expressed are those of the authors and not necessarily those of the NHS, NIHR, or the Department of Health.

## DISCLOSURES

H. U. Marschall receives consulting fees, research grants, and material support from Intercept Pharmaceuticals. L. Adorini and D. Shapiro are employees of Intercept Pharmaceuticals. None of the other authors has any conflicts of interest, financial or otherwise, to disclose.

## AUTHOR CONTRIBUTIONS

E.B. and C.W. conceived and designed research; S.M., V.N., H.M.F., J.A.K.M., A.W., E.B., and E.J. performed experiments; S.M., H.M.F., J.A.K.M., A.W., and E.J. analyzed data; S.M., L.A., D.S., P.J., J.R.M., H.-U.M., and C.W. interpreted results of experiments; S.M. and H.M.F. prepared figures; S.M. and C.W. drafted manuscript; S.M., H.M.F., J.A.K.M., A.W., E.B., L.A., D.S., P.J., J.R.M., H.-U.M., and C.W. edited and revised manuscript; S.M., V.N., H.M.F., J.A.K.M., A.W., E.B., E.J., L.A., D.S., P.J., J.R.M., H.-U.M., and C.W. approved final version of manuscript.

## References

[1] Abu-Hayyeh S, Papacleovoulou G, Lövgren-Sandblom A, Tahir M, Oduwole O, Jamaludin NA, Ravat S, Nikolova V, Chambers J, Selden C, Rees M, Marschall HU, Parker MG, Williamson C Intrahepatic cholestasis of pregnancy levels of sulfated progesterone metabolites inhibit farnesoid X receptor resulting in a cholestatic phenotype. Hepatology 57: 716–726, 2013. doi:10.1002/hep.26055. 22961653 PMC3592994

[2] Alvarez-Sola G, Uriarte I, Latasa MU, Fernandez-Barrena MG, Urtasun R, Elizalde M, Barcena-Varela M, Jiménez M, Chang HC, Barbero R, Catalán V, Rodríguez A, Frühbeck G, Gallego-Escuredo JM, Gavaldà-Navarro A, Villarroya F, Rodriguez-Ortigosa CM, Corrales FJ, Prieto J, Berraondo P, Berasain C, Avila MA Fibroblast growth factor 15/19 (FGF15/19) protects from diet-induced hepatic steatosis: development of an FGF19-based chimeric molecule to promote fatty liver regeneration. Gut 66: 1818–1828, 2017. doi:10.1136/gutjnl-2016-312975. 28119353

[3] Athukorala C, Crowther CA, Willson K; Austrailian Carbohydrate Intolerance Study in Pregnant Women (ACHOIS) Trial Group Women with gestational diabetes mellitus in the ACHOIS trial: risk factors for shoulder dystocia. Aust N Z J Obstet Gynaecol 47: 37–41, 2007. doi:10.1111/j.1479-828X.2006.00676.x. 17261098

[4] Baghdasaryan A, Claudel T, Gumhold J, Silbert D, Adorini L, Roda A, Vecchiotti S, Gonzalez FJ, Schoonjans K, Strazzabosco M, Fickert P, Trauner M Dual farnesoid X receptor/TGR5 agonist INT-767 reduces liver injury in the *Mdr2^-/-^* (*Abcb4^-/-^*) mouse cholangiopathy model by promoting biliary HCO^−^_3_ output. Hepatology 54: 1303–1312, 2011. doi:10.1002/hep.24537. 22006858 PMC3744065

[5] Baines SD, O’Connor R, Saxton K, Freeman J, Wilcox MH Comparison of oritavancin versus vancomycin as treatments for clindamycin-induced Clostridium difficile PCR ribotype 027 infection in a human gut model. J Antimicrob Chemother 62: 1078–1085, 2008. doi:10.1093/jac/dkn358. 18772161

[6] Bellamy L, Casas JP, Hingorani AD, Williams D Type 2 diabetes mellitus after gestational diabetes: a systematic review and meta-analysis. Lancet 373: 1773–1779, 2009. doi:10.1016/S0140-6736(09)60731-5. 19465232

[7] Black MH, Sacks DA, Xiang AH, Lawrence JM Clinical outcomes of pregnancies complicated by mild gestational diabetes mellitus differ by combinations of abnormal oral glucose tolerance test values. Diabetes Care 33: 2524–2530, 2010. doi:10.2337/dc10-1445. 20843973 PMC2992182

[8] Caja S, Puerta M White adipose tissue production and release of IL-6 and TNF-α do not parallel circulating and cerebrospinal fluid concentrations in pregnant rats. Horm Metab Res 40: 375–380, 2008. doi:10.1055/s-2008-1062701. 18401835

[9] Cariou B, van Harmelen K, Duran-Sandoval D, van Dijk TH, Grefhorst A, Abdelkarim M, Caron S, Torpier G, Fruchart JC, Gonzalez FJ, Kuipers F, Staels B The farnesoid X receptor modulates adiposity and peripheral insulin sensitivity in mice. J Biol Chem 281: 11039–11049, 2006. doi:10.1074/jbc.M510258200. 16446356

[10] de Castro J, Sevillano J, Marciniak J, Rodriguez R, González-Martín C, Viana M, Eun-suk OH, de Mouzon SH, Herrera E, Ramos MP Implication of low level inflammation in the insulin resistance of adipose tissue at late pregnancy. Endocrinology 152: 4094–4105, 2011. doi:10.1210/en.2011-0068. 21914778 PMC3198999

[11] De Francesco PN, Cornejo MP, Barrile F, García Romero G, Valdivia S, Andreoli MF, Perello M Inter-individual variability for high fat diet consumption in inbred C57BL/6 mice. Front Nutr 6: 67, 2019. doi:10.3389/fnut.2019.00067. 31143766 PMC6520645

[12] de Wit NJ, Bosch-Vermeulen H, de Groot PJ, Hooiveld GJ, Bromhaar MM, Jansen J, Müller M, van der Meer R The role of the small intestine in the development of dietary fat-induced obesity and insulin resistance in C57BL/6J mice. BMC Med Genomics 1: 14, 2008. doi:10.1186/1755-8794-1-14. 18457598 PMC2396659

[13] DeSisto CL, Kim SY, Sharma AJ Prevalence estimates of gestational diabetes mellitus in the United States, Pregnancy Risk Assessment Monitoring System (PRAMS), 2007-2010. Prev Chronic Dis 11: 130415, 2014. doi:10.5888/pcd11.130415. 24945238 PMC4068111

[14] Dong B, Young M, Liu X, Singh AB, Liu J Regulation of lipid metabolism by obeticholic acid in hyperlipidemic hamsters. J Lipid Res 58: 350–363, 2017. doi:10.1194/jlr.M070888. 27940481 PMC5282951

[15] Dudzik D, Zorawski M, Skotnicki M, Zarzycki W, Kozlowska G, Bibik-Malinowska K, Vallejo M, García A, Barbas C, Ramos MP Metabolic fingerprint of gestational diabetes mellitus. J Proteomics 103: 57–71, 2014. doi:10.1016/j.jprot.2014.03.025. 24698665

[16] Duran-Sandoval D, Mautino G, Martin G, Percevault F, Barbier O, Fruchart JC, Kuipers F, Staels B Glucose regulates the expression of the farnesoid X receptor in liver. Diabetes 53: 890–898, 2004. doi:10.2337/diabetes.53.4.890. 15047603

[17] Eades CE, Cameron DM, Evans JMM Prevalence of gestational diabetes mellitus in Europe: A meta-analysis. Diabetes Res Clin Pract 129: 173–181, 2017. doi:10.1016/j.diabres.2017.03.030. 28531829

[18] Freeman J, Baines SD, Jabes D, Wilcox MH Comparison of the efficacy of ramoplanin and vancomycin in both in vitro and in vivo models of clindamycin-induced *Clostridium difficile* infection. J Antimicrob Chemother 56: 717–725, 2005. doi:10.1093/jac/dki321. 16143709

[19] Gao J, Xu B, Zhang X, Cui Y, Deng L, Shi Z, Shao Y, Ding M Association between serum bile acid profiles and gestational diabetes mellitus: A targeted metabolomics study. Clin Chim Acta 459: 63–72, 2016. doi:10.1016/j.cca.2016.05.026. 27246871

[20] Garcia-Vargas L, Addison SS, Nistala R, Kurukulasuriya D, Sowers JR Gestational diabetes and the offspring: implications in the development of the cardiorenal metabolic syndrome in offspring. Cardiorenal Med 2: 134–142, 2012. doi:10.1159/000337734. 22851962 PMC3376343

[21] Glastras SJ, Tsang M, Teh R, Chen H, McGrath RT, Zaky AA, Pollock CA, Saad S Maternal obesity promotes diabetic nephropathy in rodent offspring. Sci Rep 6: 27769, 2016. doi:10.1038/srep27769. 27277011 PMC4899795

[22] Haczeyni F, Poekes L, Wang H, Mridha AR, Barn V, Geoffrey Haigh W, Ioannou GN, Yeh MM, Leclercq IA, Teoh NC, Farrell GC Obeticholic acid improves adipose morphometry and inflammation and reduces steatosis in dietary but not metabolic obesity in mice. Obesity (Silver Spring) 25: 155–165, 2017. doi:10.1002/oby.21701. 27804232 PMC5849463

[23] HAPO Study Cooperative Research Group Hyperglycemia and adverse pregnancy outcomes. N Engl J Med 358: 1991–2002, 2008. doi:10.1056/NEJMoa0707943. 18463375

[24] Holemans K, Caluwaerts S, Poston L, Van Assche FA Diet-induced obesity in the rat: a model for gestational diabetes mellitus. Am J Obstet Gynecol 190: 858–865, 2004. doi:10.1016/j.ajog.2003.09.025. 15042025

[25] Hou W, Meng X, Zhao W, Pan J, Tang J, Huang Y, Tao M, Liu F, Jia W Elevated first-trimester total bile acid is associated with the risk of subsequent gestational diabetes. Sci Rep 6: 34070, 2016. doi:10.1038/srep34070. 27667090 PMC5036171

[26] Jones HN, Woollett LA, Barbour N, Prasad PD, Powell TL, Jansson T High-fat diet before and during pregnancy causes marked up-regulation of placental nutrient transport and fetal overgrowth in C57/BL6 mice. FASEB J 23: 271–278, 2009. doi:10.1096/fj.08-116889. 18827021 PMC2626621

[27] Kalaany NY, Mangelsdorf DJ LXRS and FXR: the yin and yang of cholesterol and fat metabolism. Annu Rev Physiol 68: 159–191, 2006. doi:10.1146/annurev.physiol.68.033104.152158. 16460270

[28] Kamimae-Lanning AN, Krasnow SM, Goloviznina NA, Zhu X, Roth-Carter QR, Levasseur PR, Jeng S, McWeeney SK, Kurre P, Marks DL Maternal high-fat diet and obesity compromise fetal hematopoiesis. Mol Metab 4: 25–38, 2015. doi:10.1016/j.molmet.2014.11.001. 25685687 PMC4314531

[29] Kaufmann RC, Amankwah KS, Dunaway G, Maroun L, Arbuthnot J, Roddick JW Jr An animal model of gestational diabetes. Am J Obstet Gynecol 141: 479–482, 1981. doi:10.1016/S0002-9378(15)33263-4. 7294072

[30] King V, Dakin RS, Liu L, Hadoke PW, Walker BR, Seckl JR, Norman JE, Drake AJ Maternal obesity has little effect on the immediate offspring but impacts on the next generation. Endocrinology 154: 2514–2524, 2013. doi:10.1210/en.2013-1013. 23696566

[31] Liang C, DeCourcy K, Prater MR High-saturated-fat diet induces gestational diabetes and placental vasculopathy in C57BL/6 mice. Metabolism 59: 943–950, 2010. doi:10.1016/j.metabol.2009.10.015. 20022072

[32] Ma K, Saha PK, Chan L, Moore DD Farnesoid X receptor is essential for normal glucose homeostasis. J Clin Invest 116: 1102–1109, 2006. doi:10.1172/JCI25604. 16557297 PMC1409738

[33] Maneschi E, Vignozzi L, Morelli A, Mello T, Filippi S, Cellai I, Comeglio P, Sarchielli E, Calcagno A, Mazzanti B, Vettor R, Vannelli GB, Adorini L, Maggi M FXR activation normalizes insulin sensitivity in visceral preadipocytes of a rabbit model of MetS. J Endocrinol 218: 215–231, 2013. doi:10.1530/JOE-13-0109. 23750014

[34] Martineau M, Raker C, Powrie R, Williamson C Intrahepatic cholestasis of pregnancy is associated with an increased risk of gestational diabetes. Eur J Obstet Gynecol Reprod Biol 176: 80–85, 2014. doi:10.1016/j.ejogrb.2013.12.037. 24462052

[35] McDonald JAK, Mullish BH, Pechlivanis A, Liu Z, Brignardello J, Kao D, Holmes E, Li JV, Clarke TB, Thursz MR, Marchesi JR Inhibiting growth of *Clostridioides difficile* by restoring valerate, produced by the intestinal microbiota. Gastroenterology 155: 1495–1507.e15, 2018. doi:10.1053/j.gastro.2018.07.014. 30025704 PMC6347096

[36] Milona A, Owen BM, Cobbold JF, Willemsen EC, Cox IJ, Boudjelal M, Cairns W, Schoonjans K, Taylor-Robinson SD, Klomp LW, Parker MG, White R, van Mil SW, Williamson C Raised hepatic bile acid concentrations during pregnancy in mice are associated with reduced farnesoid X receptor function. Hepatology 52: 1341–1349, 2010. doi:10.1002/hep.23849. 20842631

[37] Milona A, Owen BM, van Mil S, Dormann D, Mataki C, Boudjelal M, Cairns W, Schoonjans K, Milligan S, Parker M, White R, Williamson C The normal mechanisms of pregnancy-induced liver growth are not maintained in mice lacking the bile acid sensor Fxr. Am J Physiol Gastrointest Liver Physiol 298: G151–G158, 2010. doi:10.1152/ajpgi.00336.2009. 19815629 PMC2822506

[38] Morrison MC, Verschuren L, Salic K, Verheij J, Menke A, Wielinga PY, Iruarrizaga-Lejarreta M, Gole L, Yu WM, Turner S, Caspers MPM, Martínez-Arranz I, Pieterman E, Stoop R, van Koppen A, van den Hoek AM, Mato JM, Hanemaaijer R, Alonso C, Kleemann R Obeticholic acid modulates serum metabolites and gene signatures characteristic of human NASH and attenuates inflammation and fibrosis progression in Ldlr-/-.Leiden mice. Hepatol Commun 2: 1513–1532, 2018. doi:10.1002/hep4.1270. 30556039 PMC6287481

[39] Moscovitz JE, Kong B, Buckley K, Buckley B, Guo GL, Aleksunes LM Restoration of enterohepatic bile acid pathways in pregnant mice following short term activation of Fxr by GW4064. Toxicol Appl Pharmacol 310: 60–67, 2016. doi:10.1016/j.taap.2016.08.021. 27609522 PMC5064858

[40] Moscovitz JE, Yarmush G, Herrera-Garcia G, Guo GL, Aleksunes LM Differential regulation of intestinal efflux transporters by pregnancy in mice. Xenobiotica 47: 989–997, 2017. doi:10.1080/00498254.2016.1250292. 28043194 PMC5495628

[41] Mudaliar S, Henry RR, Sanyal AJ, Morrow L, Marschall HU, Kipnes M, Adorini L, Sciacca CI, Clopton P, Castelloe E, Dillon P, Pruzanski M, Shapiro D Efficacy and safety of the farnesoid X receptor agonist obeticholic acid in patients with type 2 diabetes and nonalcoholic fatty liver disease. Gastroenterology 145: 574–82.e1, 2013. doi:10.1053/j.gastro.2013.05.042. 23727264

[42] Neuschwander-Tetri BA, Loomba R, Sanyal AJ, Lavine JE, Van Natta ML, Abdelmalek MF, Chalasani N, Dasarathy S, Diehl AM, Hameed B, Kowdley KV, McCullough A, Terrault N, Clark JM, Tonascia J, Brunt EM, Kleiner DE, Doo E; NASH Clinical Research Network Farnesoid X nuclear receptor ligand obeticholic acid for non-cirrhotic, non-alcoholic steatohepatitis (FLINT): a multicentre, randomised, placebo-controlled trial. Lancet 385: 956–965, 2015. doi:10.1016/S0140-6736(14)61933-4. 25468160 PMC4447192

[43] Nikolova V, Papacleovoulou G, Bellafante E, Borges Manna L, Jansen E, Baron S, Abu-Hayyeh S, Parker M, Williamson C Changes in LXR signaling influence early-pregnancy lipogenesis and protect against dysregulated fetoplacental lipid homeostasis. Am J Physiol Endocrinol Metab 313: E463–E472, 2017. doi:10.1152/ajpendo.00449.2016. 28420650 PMC5689017

[44] Ovadia C, Perdones-Montero A, Spagou K, Smith A, Sarafian MH, Gomez-Romero M, Bellafante E, Clarke LC, Sadiq F, Nikolova V, Mitchell A, Dixon PH, Santa-Pinter N, Wahlström A, Abu-Hayyeh S, Walters JR, Marschall HU, Holmes E, Marchesi JR, Williamson C Enhanced microbial bile acid deconjugation and impaired ileal uptake in pregnancy repress intestinal regulation of bile acid synthesis. Hepatology 70: 276–293, 2019. doi:10.1002/hep.30661. 30983011 PMC6619257

[45] Ovesen PG, Jensen DM, Damm P, Rasmussen S, Kesmodel US Maternal and neonatal outcomes in pregnancies complicated by gestational diabetes. a nation-wide study. J Matern Fetal Neonatal Med 28: 1720–1724, 2015. doi:10.3109/14767058.2014.966677. 25228278

[46] Pathak P, Liu H, Boehme S, Xie C, Krausz KW, Gonzalez F, Chiang JYL Farnesoid X receptor induces Takeda G-protein receptor 5 cross-talk to regulate bile acid synthesis and hepatic metabolism. J Biol Chem 292: 11055–11069, 2017. doi:10.1074/jbc.M117.784322. 28478385 PMC5491788

[47] Pellicciari R, Fiorucci S, Camaioni E, Clerici C, Costantino G, Maloney PR, Morelli A, Parks DJ, Willson TM 6α-ethyl-chenodeoxycholic acid (6-ECDCA), a potent and selective FXR agonist endowed with anticholestatic activity. J Med Chem 45: 3569–3572, 2002. doi:10.1021/jm025529g. 12166927

[48] Ryckman KK, Spracklen CN, Smith CJ, Robinson JG, Saftlas AF Maternal lipid levels during pregnancy and gestational diabetes: a systematic review and meta-analysis. BJOG 122: 643–651, 2015. doi:10.1111/1471-0528.13261. 25612005

[49] Sayin SI, Wahlström A, Felin J, Jäntti S, Marschall HU, Bamberg K, Angelin B, Hyötyläinen T, Orešič M, Bäckhed F Gut microbiota regulates bile acid metabolism by reducing the levels of tauro-beta-muricholic acid, a naturally occurring FXR antagonist. Cell Metab 17: 225–235, 2013. doi:10.1016/j.cmet.2013.01.003. 23395169

[50] Sinal CJ, Tohkin M, Miyata M, Ward JM, Lambert G, Gonzalez FJ Targeted disruption of the nuclear receptor FXR/BAR impairs bile acid and lipid homeostasis. Cell 102: 731–744, 2000. doi:10.1016/S0092-8674(00)00062-3. 11030617

[51] Song X, Vasilenko A, Chen Y, Valanejad L, Verma R, Yan B, Deng R Transcriptional dynamics of bile salt export pump during pregnancy: mechanisms and implications in intrahepatic cholestasis of pregnancy. Hepatology 60: 1993–2007, 2014. doi:10.1002/hep.27171. 24729004 PMC4194188

[52] Tobias DK, Stuart JJ, Li S, Chavarro J, Rimm EB, Rich-Edwards J, Hu FB, Manson JE, Zhang C Association of history of gestational diabetes with long-term cardiovascular disease risk in a large prospective cohort of US women. JAMA Intern Med 177: 1735–1742, 2017. doi:10.1001/jamainternmed.2017.2790. 29049820 PMC5820722

[53] Trabelsi MS, Daoudi M, Prawitt J, Ducastel S, Touche V, Sayin SI, Perino A, Brighton CA, Sebti Y, Kluza J, Briand O, Dehondt H, Vallez E, Dorchies E, Baud G, Spinelli V, Hennuyer N, Caron S, Bantubungi K, Caiazzo R, Reimann F, Marchetti P, Lefebvre P, Bäckhed F, Gribble FM, Schoonjans K, Pattou F, Tailleux A, Staels B, Lestavel S Farnesoid X receptor inhibits glucagon-like peptide-1 production by enteroendocrine L cells. Nat Commun 6: 7629, 2015. doi:10.1038/ncomms8629. 26134028 PMC4579574

[54] Van den Abbeele P, Grootaert C, Marzorati M, Possemiers S, Verstraete W, Gérard P, Rabot S, Bruneau A, El Aidy S, Derrien M, Zoetendal E, Kleerebezem M, Smidt H, Van de Wiele T Microbial community development in a dynamic gut model is reproducible, colon region specific, and selective for *Bacteroidetes* and *Clostridium* cluster IX. Appl Environ Microbiol 76: 5237–5246, 2010. doi:10.1128/AEM.00759-10. 20562281 PMC2916472

[55] Vignozzi L, Morelli A, Filippi S, Comeglio P, Chavalmane AK, Marchetta M, Toce M, Yehiely-Cohen R, Vannelli GB, Adorini L, Maggi M Farnesoid X receptor activation improves erectile function in animal models of metabolic syndrome and diabetes. J Sex Med 8: 57–77, 2011. doi:10.1111/j.1743-6109.2010.02073.x. 20955313

[56] Wahlström A, Sayin SI, Marschall HU, Bäckhed F Intestinal crosstalk between bile acids and microbiota and its impact on host metabolism. Cell Metab 24: 41–50, 2016. doi:10.1016/j.cmet.2016.05.005. 27320064

[57] Walters JR, Johnston IM, Nolan JD, Vassie C, Pruzanski ME, Shapiro DA The response of patients with bile acid diarrhoea to the farnesoid X receptor agonist obeticholic acid. Aliment Pharmacol Ther 41: 54–64, 2015. doi:10.1111/apt.12999. 25329562

[58] Wang D, Zhu W, Li J, An C, Wang Z Serum concentrations of fibroblast growth factors 19 and 21 in women with gestational diabetes mellitus: association with insulin resistance, adiponectin, and polycystic ovary syndrome history. PLoS One 8: e81190, 2013. doi:10.1371/journal.pone.0081190. 24260557 PMC3834317

[59] Wang YD, Chen WD, Wang M, Yu D, Forman BM, Huang W Farnesoid X receptor antagonizes nuclear factor κB in hepatic inflammatory response. Hepatology 48: 1632–1643, 2008. doi:10.1002/hep.22519. 18972444 PMC3056574

[60] White SL, Pasupathy D, Sattar N, Nelson SM, Lawlor DA, Briley AL, Seed PT, Welsh P, Poston L; UPBEAT Consortium Metabolic profiling of gestational diabetes in obese women during pregnancy. Diabetologia 60: 1903–1912, 2017. doi:10.1007/s00125-017-4380-6. 28766127 PMC6448883

[61] Wikström Shemer E, Marschall HU, Ludvigsson JF, Stephansson O Intrahepatic cholestasis of pregnancy and associated adverse pregnancy and fetal outcomes: a 12-year population-based cohort study. BJOG 120: 717–723, 2013. doi:10.1111/1471-0528.12174. 23418899

[62] Xu Y, Li F, Zalzala M, Xu J, Gonzalez FJ, Adorini L, Lee YK, Yin L, Zhang Y Farnesoid X receptor activation increases reverse cholesterol transport by modulating bile acid composition and cholesterol absorption in mice. Hepatology 64: 1072–1085, 2016. doi:10.1002/hep.28712. 27359351 PMC5033696

[63] Zhang L, Sugiyama T, Murabayashi N, Umekawa T, Ma N, Kamimoto Y, Ogawa Y, Sagawa N The inflammatory changes of adipose tissue in late pregnant mice. J Mol Endocrinol 47: 157–165, 2011. doi:10.1530/JME-11-0030. 21697073 PMC3162642

[64] Zhang Y, Lee FY, Barrera G, Lee H, Vales C, Gonzalez FJ, Willson TM, Edwards PA Activation of the nuclear receptor FXR improves hyperglycemia and hyperlipidemia in diabetic mice. Proc Natl Acad Sci USA 103: 1006–1011, 2006. doi:10.1073/pnas.0506982103. 16410358 PMC1347977

